# Application of 3D Computed Tomography Reconstruction Images to Assess the Thickness and Dimensions of the Posterior Palatal Seal Area

**DOI:** 10.1155/2019/7912371

**Published:** 2019-02-19

**Authors:** Soeun Lim, Seoung-Jin Hong, Joo-Young Ohe, Janghyun Paek

**Affiliations:** ^1^Department of Prosthodontics, Kangwon National University Hospital, Chuncheon 24289, Republic of Korea; ^2^Department of Prosthodontics, Kyung Hee University Dental Hospital, Seoul 02447, Republic of Korea; ^3^Department of Oral and Maxillofacial Surgery, School of Dentistry, Kyung Hee University, Seoul 02447, Republic of Korea; ^4^Department of Prosthodontics, School of Dentistry, Kyung Hee University, Seoul 02447, Republic of Korea

## Abstract

Few studies have been reported on the scientific measurements of the thickness and dimensions of the posterior palatal seal (PPS) area. The purpose of this study is to measure and analyze the thickness of palatal mucosa by using a three-dimensional (3D) model reconstructed with computed tomography (CT) images and to present objective values by identifying the PPS area. The CT images were reconstructed as a 3D model by separating the maxillary palate mucosa and teeth. Each reconstructed model was analyzed and the thickness was measured at 93 crossing points of each divided plane. The dimension of the PPS area was measured and the right and left dimensions of the PPS area were compared. The thickness of the palatal mucosa was thicker toward the posterior area. The thickness increased in the lateral direction and decreased again. In the PPS area, the mean dimension between the rearmost of anterior border and the most posterior line was 2.19 mm and the mean dimension between the forefront of anterior border and the most posterior line was 5.19 mm in the right side and 5.16 mm in the left side. The mean dimension from the center of the palate to the right most forward point was 6.85 mm, and the left was 7.36 mm. The new measurement method of palatal mucosal thickness is noninvasive, accurate, and easy to store and study, so it can be used effectively in planning and manufacturing the maxillary complete denture in the digital workflows.

## 1. Introduction

Complete denture treatment is a complex and challenging procedure that involves the design of the prosthesis. This prosthesis is placed in the midst of a vital, dynamic oral environment on a foundation that is very unstable. Therefore, clinicians must assure adequate retention, support, and stability to achieve optimal results for complete denture treatments [1–3].

In routine clinical practice, border molding of the oral cavity is required to obtain maximum extension of the complete denture. For the preparation of the maxillary complete denture, the posterior border should be determined, and the sealing of the posterior area should be performed [4]. A change in ambient pressure causes the amount of air in the film of saliva, and it affects the retention of the complete denture. A tissue of the posterior palatal seal (PPS) area has functional movement, and, during processing of the denture, a gap between the tissue and the intaglio surface of the denture is caused by the polymerization of the acrylic resin, mostly in the PPS area. Therefore, it is important to seal hermetically in the PPS area for the retention of the maxillary complete denture [5]. The PPS area can usually be extended forward about 4mm from the distal border of the denture. The seal may be a little wider than this in some areas, while in the area where it passes through the hamular notch, it will narrow to only 2 mm in width [6]. Silverman [7] performed a study on 92 patients to clinically, radiographically, and histologically evaluate the PPS area and reported that the greatest mean anteroposterior width of the PPS area was 8.0mm (range 5-12mm). The mean width was found to be different for the right (8.2mm) and left sides (8.1mm).

Considering these dimensional measurements, additional dental compound impression material can be added across the hamular notches and palate during border molding. Post damming is established as a part of a total peripheral seal, not a separate entity, by pressing the posterior border with an additional compound. This procedure is known as a selective pressure impression technique [8–10]. A wide PPS area is optimal because it allows placement of a substantial seal. The depth of the PPS area is determined by the degree of vertical movement of the soft palate, and the degree of vertical movement of the soft palate is influenced by the thickness of soft palate. The thicker the soft palate, the more vertical the movement of the soft palate [11]. However, the post damming is performed empirically by clinicians. To date, few studies have been reported on the scientific measurements of the thickness and dimensions of the PPS area.

Data about the exact dimensions and thickness of the PPS area allows the post damming to be performed with more specific guidelines. The purpose of this study was to assess and measure the dimensions and thickness of the palatal mucosa of the oral cavity in a systematic manner using reconstructed three-dimensional (3D) model to objectively determine the dimensions of the PPS area.

## 2. Materials and Methods

The present study was approved by the Institutional Review Board (KHD IRB 1712-2).

### 2.1. Subjects

Retrospective analysis was performed using computed tomography (CT) images taken at the Dental Hospital from July 2012 to October 2017. A total of 54 patients underwent CT with open mouth, and 34 patients were excluded due to the following conditions: (1) 8 cases with abnormal maxillary deformations, (2) 24 cases that experienced difficulties in separating the palatal mucosa from the surrounding soft tissue, and (3) 2 cases with severe metal artifacts due to the dental prosthesis. Consequently, the study was performed on CT images of 20 subjects (11 males and 9 females; 21 to 80 years of age).

### 2.2. CT Device

The high-resolution 128-slice multidetector computed tomography (MDCT) (Ingenuity Core 128; Philips Medical Systems, Best, The Netherlands) and 64-slice MDCT (Aquilion Prime Model TSX-303A; Toshiba Medical Systems Corp., Tokyo, Japan) scanners were employed with an axial slice thickness of 0.5-1.0 mm. Anatomic modeling requires selection of a defined Hounsfield unit (HU) from CT data that establishes a threshold for accurate modeling of the required tissue type.

### 2.3. 3D Model Reconstruction from CT Image

**Figure 1:**
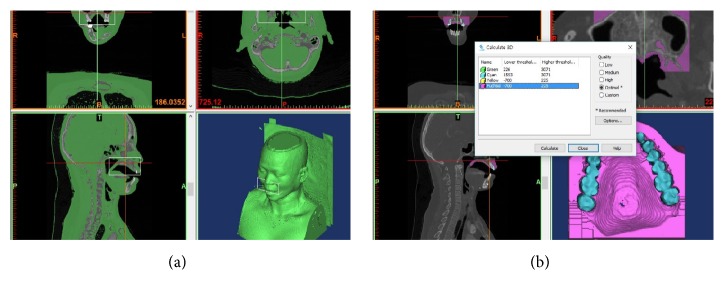
Two-dimensional cross-sectional CT images were stacked, reconstructed 3D model, and segmented palatal mucosa and teeth model. (a) Reconstructed 3D model. (b) Palatal mucosa and teeth model.

The CT images of the patients were analyzed and Mimics software (ver. 19.0; Materialise, Leuven, Belgium) was used to generate computerized composite palate models of the patients, including the soft tissue and dentition. To reconstruct data in Mimics software, all of the CT images were stored in digital imaging and communications in medicine (DICOM) file format, and a set of stacked two-dimensional cross-sectional images was imported (Figure 1(a)). A segmentation was applied to each 3D model to identify and separate the palatal mucosa from the surrounding tissues. Predefined settings of the Hounsfield range were established for the density of the biological tissue in which a lower threshold allows segmentation of soft tissue, whereas a higher threshold allows segmentation of bone. A thresholding allows soft tissue to easily be separated from bone. After classifying the pixels, a segmentation was performed to reconstruct the palate mucosa and teeth 3D model by removing unnecessary images from a mask (Figure 1(b)).

### 2.4. Measurement of the Posterior Palatal Mucosa Thickness

**Figure 2:**
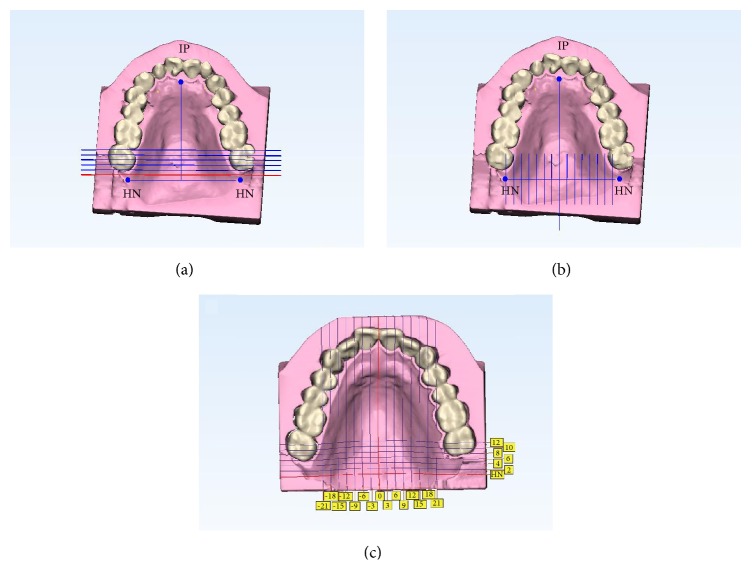
Model was divided and thickness of mucosa was measured at 93 points. (a) Seven sections parallel to coronal plane at 2mm intervals from HN line. (b) 15 sections parallel to sagittal plane at 3mm intervals from mid-palatal line to both sides. (c) 93 crossing points of each divided plane. IP, incisive papilla; HN, hamular notch.

The reconstructed palate 3D models were calibrated using 3-matic software (ver. 11.0; Materialise, Leuven, Belgium). The intersection point of the vertical line drawn at the incisive papilla on the line connecting both hamular notches is defined as the center point. Using the above four reference points, three planes perpendicular to each other are formed passing through the center point and referred to as reference planes of the coronal plane, sagittal plane, and horizontal plane. In coronal plane, all models were divided into 2 mm intervals forward from the hamular notch line parallel to the coronal plane, and 7 sections were measured (Figure 2(a)). In sagittal plane, 3 mm intervals to both sides from the mid-plate parallel to the sagittal plane and 15 sections were measured (Figure 2(b)). The thickness was measured at 93 crossing points of each divided plane (Figure 2(c)).

### 2.5. Dimensions of the Posterior Palatal Seal Area

**Figure 3:**
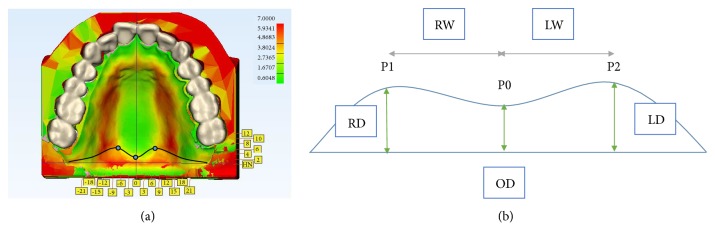
Thickness of mucosa was measured and displayed in color, and the PPS area was determined. (a) Color map of thickness and the PPS area border line. (b) Diagram of vertical plane of the PPS area. P0, rearmost point of anterior border; P1, foremost point of anterior border of right side; P2, foremost point of anterior border of left side; RW, dimension between P1 and mid-palatal line; LW, dimension between P2 and mid-palatal line; OD, dimension between P0 and hamular notch line; RD, dimension between P1 and hamular notch line; LD, dimension between P2 and hamular notch line.

The PPS area was determined based on the wall thickness analysis of 3-matic software. Two points were marked at the most forefront points on both sides of the anterior border of the red region, and one point was marked at the rearmost point of the anterior border of the red region. The red color indicated greater thickness compared with other areas. The PPS area dimensions were measured between these 3 points, in addition to the coronal and sagittal plane (Figure 3).

### 2.6. Statistical Analysis

Statistical analysis was performed using IBM SPSS software (ver. 23.0; IBM SPSS Inc., Chicago, IL, USA) for WIN. All data were subjected to normality and homogeneity of variance on a Shapiro-Wilk test for each group. The t-test was used to examine the significant difference between the right and left dimensions of the PPS area. The significance level was set at 5%.

## 3. Results

### 3.1. Thickness of the Posterior Palatal Mucosa

**Figure 4:**
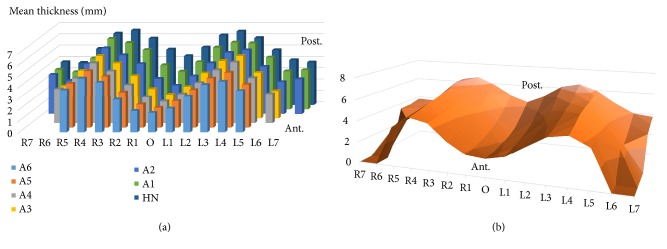
Mean thickness value of mucosa at measured points. (a) 3D column chart. (b) 3D surface chart. O, mid-palatal plane; R1-17, and L1-L7, sectioned plane; HN, hamular notch line; Ant., anterior direction; Post, posterior direction.

The mean thickness values were ranged from 1.71 mm to 6.59 mm and varied according to the PPS region. The thickness value was gradually thickened toward the posterior border of the PPS area and the anterior to posterior variation of the thickness decreased gradually from the center of the palate toward the lateral side. The thickness value was the smallest in the mid-palatal area. The thickness value was increased in the lateral direction and the largest values of the right and left sides were 9 mm from the center of the palate of the most posterior line of the PPS area; after that, it decreased again (Table 1, Figure 4).

### 3.2. Dimensions of the Posterior Palatal Seal Area

The mean dimension between the rearmost of anterior border and the hamular notch plane in the PPS area was 2.19 mm and the mean dimension between the forefront of anterior border and the hamular notch plane was 5.19 mm in the right side and 5.16 mm in the left side. There was no significant difference between the right and left sides (p>0.05). The mean dimension from the mid-palatal plane to the right most forward point was 6.85 mm, and the left was 7.36 mm and there was no significant difference between both sides (p>0.05) (Table 2).

## 4. Discussion

Retention is essential for the successful treatment of the maxillary complete denture. The retention of the denture has been defined as the resistance to vertical movement away from the tissues [12] and as a quality that is inherent in the prosthesis to resist dislodgement forces along the path of insertion [13]. It is important to discuss and identify factors associated with the retention of the complete denture, the importance of the PPS, its location, design, placement, and influence on processing.

The PPS area is the portion of the intaglio surface of a maxillary removable complete denture located at the posterior border, which places pressure, within physiologic limits, on the PPS area of the soft palate; this seal ensures intimate contact of the denture base to the soft palate and improves the retention of the denture [13]. The importance and function of the PPS cannot be overemphasized in successful maxillary complete dentures. The contact was maintained by sealing between the denture and soft tissue during functional movements of the stomatognathic system, which then decreases the gag reflex. The food accumulation was decreased by sealing with adequate tissue compressibility and also patient discomfort was decreased when the tongue contact on the posterior part of the denture. The PPS not only increases retention and stability by making a partial vacuum, but also compensates for the volumetric shrinkage that occurred during polymethyl methacrylate polymerization. Finally, the strength of the denture base was increased by the PPS [5, 14, 15].

The dimensions and thickness were measured in this study to provide a more robust assessment of the PPS applications. Many studies have demonstrated the possibility of using images to measure the thickness of the palatal masticatory mucosa with conventional CT, cone beam computed tomography (CBCT), and ultrasonic devices [16–18]. Compared to conventional CT, CBCT presents advantages such as lower radiation, greater comfort for the patient, and lower costs. However, due to the low contrast resolution in CBCT scans, data from CBCT studies are less reliable for soft tissue measurement [19]. Histograms of MDCT data reveal distinct peaks that generally correspond to hard and soft tissue types, whereas CBCT plots revealed a more diffuse appearance. Segmentation of CT data is a necessary step for creating anatomical models. Pixel thresholding, often based on peaks seen in pixel histograms, is a typical method for segmentation of bone in CT scans. The lack of distinct peaks in CBCT histograms compared to MDCT data indicates less contrast resolution in CBCT scans [19]. Data from MDCT studies is acceptable as a threshold for anatomic model output. Ultrasonic devices were reported to have highly accurate measurements, showing a correlation coefficient of 0.921. However, the reliability of the measurement was relatively low (measurement error: 0.54) [20]. Although the ultrasonic device was noninvasive and easily applicable, this method is technique sensitive, has low reproducibility, and has limitations in sites with a palatal vault depth [21]. This study used conventional CT data, which provide measurements of the posterior palatal mucosa with no limitation due to anatomical structure and have high accuracy, better quality of reconstructed 3D images, and ease of standardization.

In previous studies, the palatal mucosa thickness was measured comparing to the tooth. However, for the mucosal thickness measurement research of edentulous patients, it is necessary to measure using anatomical structures other than teeth as indicators. In this study, the thickness was measured according to the distance from the midline and the hamular notch line, and the mean thickness of the palatal mucosa varied according to region of the PPS area. The value gradually thickened toward the posterior border of the PPS area. The anterior to posterior variation in thickness decreased with distance from the mid-palatal line. The boundary of the hard palate at the junction of the hard and soft palates is typically butterfly-shaped. The point at which the soft palate meets the hard palate is more forefront at the lateral side of the palate rather than at the palatal midline [22]. This anatomical feature provides an explanation for the anteroposterior mucosal thickness variation of the posterior palate relatively large increasing at the center of the palate compared to the lateral sides.

The average anterior to posterior dimension of the PPS area was from 2 mm to 5 mm. Narvekar and Appelbaum [19] reported that, in an ultrasonographic study of 15 participants, the average range for the anterior to posterior dimension of the PPS area was estimated to be between 4 mm and 6 mm. According to Hardy and Kapur [6], the PPS area can usually be extended forward about 4 mm from the posterior border of the denture, and Silverman [7] found that the greatest mean of the anteroposterior width of the PPS area is 8 mm (with 5-12 mm range). The differences in the dimension of the PPS area reported in various studies may be attributed to several factors. First, different anatomical references and methods were used to identify the anterior and posterior borders of the PPS area. Additionally, in studies that used vibrating lines to determine the PPS area, consistently marking the vibrating line would be difficult.

Digital technology has been adopted in clinical dentistry, and the digital impression technique has more recently been applied for fabricating various types of prostheses [23, 24]. Fixed prostheses and implant prostheses can now be created without conventional impression materials and casts. Intraoral scanning of the edentulous ridges has also been studied [25, 26]. However, there are limitations t fabricating retentive and supportive complete dentures with only the intraoral scanning of the edentulous area. Intraoral scanning can be performed only via a nonpressure impression technique. Unlike fixed prosthesis, the selective pressure technique should be applied to create a complete denture. Based on this study, it is possible to selectively pressure the PPS area by superimposing reconstructed 3D model and intraoral scanning data of the maxillary edentulous arches based on radiopaque resin marker [25]. Therefore, by accurate analysis of the dimension and thickness of the PPS area, clinicians can perform nonpressure intraoral scanning and revise the scan data to selectively press the palatal mucosa. Also, incorporating into digital workflows and combining it with other digital devices like intraoral scanner, more accurate and clinically effective digital complete denture can be manufactured.

## 5. Conclusions

The thickness of the palatal mucosa was thicker toward the posterior area and increased in the lateral direction and decreased again. The anterior border of the PPS area was a butterfly shape and there was no significant difference between the right and left side dimensions of the PPS area.

A new noninvasive method to consistently obtain images and measurements of the posterior palatal mucosa is described. This reliable and reproducible method could be beneficial for analyzing soft tissue and can be used to plan and fabricate the maxillary complete denture in digital workflow.

## Figures and Tables

**Table 1:** Mean thickness value (±SD) of posterior palatal mucosa (mm).

	R7	R6	R5	R4	R3	R2	R1	O	L1	L2	L3	L4	L5	L6	L7
A6			3.71 (0.80)	4.76 (0.78)	4.40 (1.22)	2.89 (1.07)	1.87 (0.79)	1.71 (0.59)	2.09 (0.79)	3.16 (1.01)	4.19 (1.23)	4.49 (0.64)	3.66 (0.88)		
A5			3.90 (0.99)	5.01 (0.93)	4.54 (1.14)	3.12 (0.88)	2.05 (0.83)	1.75 (0.67)	2.35 (0.82)	3.33 (1.06)	4.37 (1.26)	4.88 (0.60)	3.82 (1.03)		
A4		3.00 (1.04)	3.97 (1.22)	5.31 (1.02)	4.58 (0.98)	3.35 (0.93)	2.27 (0.91)	1.91 (0.73)	2.52 (0.99)	3.55 (1.19)	4.68 (1.29)	5.37 (0.76)	3.98 (1.08)	2.56 (1.02)	
A3		2.85 (1.40)	4.23 (1.37)	5.55 (1.01)	4.89 (1.03)	3.77 (0.99)	2.60 (1.14)	2.10 (0.89)	2.80 (1.09)	4.01 (1.06)	5.10 (0.98)	5.54 (0.76)	4.06 (1.28)	2.42 (1.06)	
A2	3.47 (1.56)	2.95 (1.38)	4.42 (1.40)	5.81 (0.86)	5.20 (1.04)	4.42 (1.10)	3.11 (1.17)	2.51 (0.95)	3.34 (1.12)	4.50 (1.08)	5.41 (1.06)	5.64 (0.68)	4.18 (1.37)	2.82 (1.18)	3.07 (1.20)
A1	3.52 (1.54)	3.31 (1.38)	4.51 (1.10)	6.25 (0.97)	5.88 (1.15)	5.26 (1.29)	3.91 (1.32)	3.36 (1.16)	4.19 (1.30)	5.47 (1.29)	5.92 (1.16)	5.85 (0.81)	4.60 (1.40)	3.37 (1.17)	3.48 (1.18)
HN	3.78 (1.45)	3.71 (1.26)	4.96 (1.20)	6.30 (1.20)	6.59 (1.34)	5.87 (1.63)	4.89 (1.62)	4.32 (1.53)	5.07 (1.68)	6.15 (1.56)	6.50 (1.14)	5.91 (1.37)	4.84 (1.38)	3.96 (1.09)	3.76 (1.35)

HN, hamular notch line; O, mid-palatal line; A1, 2 mm anterior to HN; A2, 4 mm anterior to HN; A3, 6 mm anterior to HN; A4, 8 mm anterior to HN; A5, 10 mm anterior to HN; A6, 12 mm anterior to HN; R1, 3 mm right to midline; R2, 6 mm right to midline; R3, 9 mm right to midline; R4, 12 mm right to midline; R5, 15 mm right to midline; R6, 18 mm right to midline; R7, 21 mm right to midline; L1, 3 mm left to midline; L2, 6 mm left to midline; L3, 9 mm left to midline; L4, 12 mm left to midline; L5, 15 mm left to midline; L6, 18 mm left to midline; L7, 21 mm left to midline.

**Table 2:** Mean dimension value (±SD) of right and left PPS area (mm).

	OD	RD	LD	RW	LW
Mean (±SD)	2.17 (1.17)	5.19 (1.69)^a^	5.16 (1.70)^a^	6.85 (1.68)^b^	7.36 (1.81)^b^

Same superscript letters indicate statistically similar groups (p>0.05).

OD, dimension between P0 and hamular notch line; RD, dimension between P1 and hamular notch line; LD, dimension between P2 and hamular notch line; RW, dimension between P1 and mid-palatal line; LW, dimension between P2 and mid-palatal line.

## Data Availability

The CT images and reconstructed and calibrated 3D model data used to support the findings of this study are restricted by the Institutional Review Board (KHD IRB 1712-2) in order to protect patient privacy.
